# Comparative analyses of the bacterial communities present in the spontaneously fermented milk products of Northeast India and West Africa

**DOI:** 10.3389/fmicb.2023.1166518

**Published:** 2023-10-11

**Authors:** Philippe Sessou, Santosh Keisam, Mariama Gagara, Gwladys Komagbe, Souaïbou Farougou, Jacques Mahillon, Kumaraswamy Jeyaram

**Affiliations:** ^1^Research Unit on Communicable Diseases, Laboratory of Research in Applied Biology, Polytechnic School of Abomey-Calavi, University of Abomey-Calavi, Abomey-Calavi, Cotonou, Benin; ^2^Microbial Resources Division, Institute of Bioresources and Sustainable Development (IBSD), Takyelpat Institutional Area, Imphal, Manipur, India; ^3^Central Livestock Laboratory, Niamey, Niger; ^4^Laboratory of Food and Environmental Microbiology, Earth and Life Institute, Université catholique de Louvain, Louvain-la-Neuve, Belgium; ^5^IBSD Regional Centre, Tadong, Gangtok, Sikkim, India

**Keywords:** spontaneously fermented milk products, MiSeq amplicon sequencing, *Lactobacillus*, *Lactococcus*, *Gluconobacter*, *Acetobacter*, *Macrococcus caseolyticus*, *Streptococcus infantarius*

## Abstract

**Introduction:**

Spontaneous fermentation of raw cow milk without backslopping is in practice worldwide as part of the traditional food culture, including “*Doi*” preparation in earthen pots in Northeast India, “*Kindouri*” of Niger and “*Fanire*” of Benin prepared in calabash vessels in West Africa. Very few reports are available about the differences in bacterial communities that evolved during the spontaneous mesophilic fermentation of cow milk in diverse geographical regions.

**Methods:**

In this study, we used high throughput amplicon sequencing of bacterial 16S rRNA gene to investigate 44 samples of naturally fermented homemade milk products and compared the bacterial community structure of these foods, which are widely consumed in Northeast India and Western Africa.

**Results and discussion:**

The spontaneous milk fermentation shared the lactic acid bacteria, mainly belonging to *Lactobacillaceae (Lactobacillus)* and *Streptococcaceae (Lactococcus)* in these two geographically isolated regions. Indian samples showed a high bacterial diversity with the predominance of *Acetobacteraceae (Gluconobacter and Acetobacter)* and *Leuconostoc*, whereas *Staphylococcaceae (Macrococcus)* was abundant in the West African samples. However, the *Wagashi* cheese of Benin, prepared by curdling the milk with proteolytic leaf extract of *Calotrophis procera* followed by natural fermentation, contained *Streptococcaceae (Streptococcus* spp.) as the dominant bacteria. Our analysis also detected several potential pathogens, like *Streptococcus infantarius* an emerging infectious foodborne pathogen in *Wagashi* samples, an uncultured bacterium of *Enterobacteriaceae* in *Kindouri* and *Fanire* samples, and *Clostridium* spp. in the *Doi* samples of Northeast India. These findings will allow us to develop strategies to address the safety issues related to spontaneous milk fermentation and implement technological interventions for controlled milk fermentation by designing starter culture consortiums for the sustainable production of uniform quality products with desirable functional and organoleptic properties.

## 1. Introduction

Fermented milk products are an essential component of the traditional food cultures of different ethnic communities worldwide. These fermented milk products are prepared from the raw or boiled milk of cow, buffalo, yak, camel, goat, and sheep through backslopping or spontaneous fermentation. *Kefir* of Russia, *Koumiss* and *Tarang* of Mongolia and China, *Dahi* of India, *Suero Costeno* of Colombia, and *Lait caillé* of sub-Saharan countries are a few examples of well-known traditional fermented milk products (de Melo Pereira et al., 2022). *Dahi* is an analog of *yogurt*, a semi-solid ready-to-drink Indian food generally prepared from boiled milk by backslopping a part of the previous batch of successful fermentation as a starter (Dewan and Tamang, 2007; Rai et al., 2016; Mudgal and Prajapati, 2017; Mallappa et al., 2021). Unlike thermophilic *yogurt* making, *Dahi* is usually incubated at room temperature (15–30°C) for 1–3 days in mesophilic fermentation (Mudgal and Prajapati, 2017; Mallappa et al., 2021; de Melo Pereira et al., 2022). The Mongoloid ethnic communities in Northeast India mostly prefer the spontaneous fermentation of fresh cow milk in earthen wares without backslopping (Joishy et al., 2019). The local vernacular names of these products are *Doi, Dei, Dahi*, and *Mishti-dahi* in Assam, Tripura, and Meghalaya, or *Sangom afamba* in Manipur (Joishy et al., 2019; Barooah et al., 2020; Wahengbam et al., 2020; Mallappa et al., 2021). Similarly, Fulani communities in Western African countries practice spontaneous fermentation of unpasteurised raw milk in calabash vessels without backslopping, such as *Kindirmou* in Niger and *Fanire* in Benin (Agyei et al., 2020; Fagbemigun et al., 2021). Backslopping naturally selects well-adapted microbes, resulting in consistent-quality products, whereas spontaneous fermentation results in highly heterogeneous products. Moreover, the microbes present in raw milk of animal origin also influence fermentation, often resulting in poor-quality products (Sun and D'Amico, 2021).

Recent next-generation sequencing (NGS)-based cultivation-independent studies showed a ubiquitous presence of beneficial bacteria, mainly *Lactobacillaceae* (*Lactobacillus delbrueckii, Lactobacillus kefiranofaciens, Lactobacillus helveticus*, and *Leuconostoc mesenteroides*) and *Streptococcaceae* (*Streptococcus thermophilus* and *Lactococcus lactis*) in most of the naturally fermented milk products worldwide (Jayashree et al., 2013; Oki et al., 2014; Bokulich et al., 2015; Motato et al., 2017; Shangpliang et al., 2018; Mallappa et al., 2021; de Melo Pereira et al., 2022). The NGS-based studies also highlighted the dominance of acetic acid bacteria, *Acetobacteraceae* (mainly *Gluconobacter* and *Acetobacter*), in several spontaneous fermented milk products (Shangpliang et al., 2018; de Melo Pereira et al., 2022). Among yeast, *Kluyveromyces marxianus, Geotrichum candidum*, and *Saccharomyces cerevisiae* are recorded across naturally fermented milk products (Bokulich et al., 2015; Sessou et al., 2019; Gastrow et al., 2020; Tenorio-Salgado et al., 2021). The substrate-specific adaptive evolution during backslopping of boiled milk and mesophilic fermentation shapes a particular group of bacterial communities, resulting in uniform quality products, whereas spontaneous fermentation of raw milk results in high variability in taste, flavor, and texture due to variations in geography, milk source, local climatic conditions, quality, and composition of milk (Zhong et al., 2016; Peng et al., 2021; Zhang et al., 2021; Zhao et al., 2021; Tamang, 2022). At the same time, such spontaneous fermentation also results in products displaying unique tastes and aromas, such as *Khoormog* and *Airag* of Mongolia and China (Oki et al., 2014; de Melo Pereira et al., 2022), *Churpii* of India (Shangpliang et al., 2018), and *Wagashi* cheese of Benin (Sessou et al., 2019), which deserve geographical indicator tagging. Therefore, there is a need to study the bacterial community structure and safety of these products by culture-independent NGS analysis to understand the presence/absence of beneficial microbes and unwanted potential pathogens (Tamang et al., 2021; de Melo Pereira et al., 2022; Rai and Tamang, 2022). This understanding will allow us to design starter culture consortia for sustainable industrial production of quality and safe fermented milk products with health benefits (Agyei et al., 2020).

In this study, we aimed to understand how spontaneous fermentation of raw cow milk without backslopping shapes the bacterial diversity in two geographically separated regions of two continents by analyzing homemade milk products, namely, *Doi, Sangom afamba*, and *Mishti dahi* of Northeast India; and *Kindirmou* and *Fanire* of Western Africa, by using 16S rRNA gene amplicon sequencing. *Doi* is traditionally prepared from fresh cow milk without backslapping by keeping it in an earthen pot wrapped with banana leaves and allowing it to ferment for 2 days (Joishy et al., 2019). *Sangom afamba* is also prepared from fresh cow milk, similar to *Doi*, in a traditional earthen pot, but a spoonful of the previous fermented batch is added and covered with a muslin cloth and allowed to stand for 2–3 days. In the *Mishti dahi*, cow milk is boiled with up to 10% sugar, cooled, and poured into small earthen pots. A starter from the previous batch was added and kept at room temperature for 1–2 days. Traditionally, *Doi, Sanggon afamba*, and *Misthi dahi* are consumed raw and taken along with steamed rice. *Sangom afamba* is essential for performing rituals in certain traditional religious ceremonies, such as the *na-hutpa* (ear-piercing ceremony of children) in Manipur. Fulani communities of Western African countries practice spontaneous fermentation of unpasteurised fresh cow milk without backslopping in calabash vessels for 1 day at room temperature to allow spontaneous fermentation (Sessou et al., 2019), such as *Kindirmou* in Niger and *Fanire* in Benin, which are commonly used as a dessert or refreshment by people in these countries. In addition, we studied a unique West African traditional cheese, *Wagashi*, with no report available on its associated bacterial communities. *Wagashi* (Gassire in the local Fulfulde language) is a soft, fresh cheese from Benin produced from cow milk. In the traditional preparation, 1 L of boiled cow milk is mixed with ~0.5 L of fresh milk with the extract of *Calotropis procera* leaves (10–15 g). The mixture is kept at a warm temperature (60–70°C) until coagulation is achieved, and then the curd and whey are separated. The curd portion is drained, molded without pressing, and incubated at room temperature. The pink-colored Wagashi cheese is prepared by soaking in a Sorghum (*Sorghum bicolor*) leaf extract brine for the pink color formation. Unlike conventional cheeses, *Wagashi* is prepared by curdling cow milk with proteolytic *Calotrophis procera* leaf extract (Akogou et al., 2018; Sessou et al., 2019). We compared the bacterial community structure of both uncoloured and colored *Wagashi* cheese samples from Benin using 16S rRNA gene amplicon sequencing and related its bacterial community to similar cheeses reported from earlier studies (Shangpliang et al., 2018; Zhao et al., 2021). In addition, we aimed to assess the safety of these spontaneously fermented milk products by detecting potential foodborne pathogens.

## 2. Materials and methods

### 2.1. Sampling and homogenisation

The spontaneously fermented milk product samples from Northeast India, Niger, and Benin were collected under aseptic conditions (Table 1). Samples of uncoloured and colored *Wagash*i cheese prepared traditionally at home and locally marketed in different towns in Benin were also collected. The samples were transported in ice-cooled boxes and stored in the laboratory at −80°C for further analysis. A volume of 10 mL of each fermented milk sample was homogenized with 90 mL of 2% sodium citrate solution, while 25 g of each cheese sample was homogenized with 45 mL of buffered peptone water (Bio-Rad, pH 7.0 ± 0.2) at 200 rpm for 2 min using a Stomacher 400 Circulator (Seward, United Kingdom). After allowing the large debris to settle down, the resulting clear homogenate was used for metagenomic DNA extraction.

**Table 1:** Features of naturally fermented cow milk product samples collected in Northeast India and West Africa.

**Region**	**Local name**	**Place of collection**	**Coordinates**	**Altitude (msl)**	**Temperature range (^o^C)**	**Pre-treatment**	**Backslopping**	**Number of samples**
Northeast India	*Doi*	Guwahati, Assam	26.1792° N, 91.7533° E	55	11–33	No	No	3
	*Doi*	Silchar, Assam	24.8207° N, 92.8018° E	22	11–33	No	No	2
	*Doi*	Jorhat, Assam	26.7477° N, 94.2132° E	116	11–31	No	No	3
	*Doi*	Agartala, Tripura	23.8419° N, 91.2820° E	13	10–34	No	No	4
	*Doi*	Shillong, Meghalaya	25.5811° N, 91.8865° E	1525	4–23	No	No	3
	*Sanggom afamba*	Imphal, Manipur	24.8072° N, 93.9341° E	786	5–30	No	Yes	3
	*Mishti Dahi*	Agartala, Tripura	23.8419° N, 91.2820° E	13	10–34	Addition of 10% sugar and boiling	Yes	3
West Africa	*Kindirmou*	Niger (Tillaberi, Dosso, Niamey)	14.2061° N, 1.4580° E; 13.0505° N, 3.2081° E 13.5116° N, 2.1254° E	180–228	18–40	No	No	8
	*Fanirè*	Benin (Agouna, Pehunco, Parakou Djougou, Houeyogbe)	9.3467° N, 2.6090° E 10.2283° N, 2.0019° E 9.3466° N, 2.609° E 6.5321° N, 1.8708° E	234–444	17–40	No	No	5
	Uncoloured *Wagashi* cheese	Benin (Abomey-Calavi, Cotonou)	6.3719° N, 2.4348° E 6.4503° N, 2.3468° E	51–54	24–32	Yes, boiling and *Calotropis* leaf extract addition	No	5
	Colored *Wagashi* cheese	Benin (Abomey-Calavi, Cotonou)	6.3719° N, 2.4348° E 6.4503° N, 2.3468° E	51–54	24–32	Yes, boiling, *Calotropis* leaf extract addition and Sorghum leaf extract coloration	No	5

### 2.2. Metagenomic DNA extraction

Two different extraction methods were independently used to extract DNA from fermented milk and cheese samples. Metagenomic DNA of fermented milk samples was extracted according to method V, described earlier by Keisam et al. (2016). Briefly, 1.5 mL of homogenate was transferred to a sterile 2-ml screw-cap tube containing zirconia/silica beads and centrifuged. The resulting pellets were treated with enzymes (50 KU lysozyme and 25 U mutanolysin) and incubated at 37°C for 1 h, incubated with proteinase-K at 65°C for 1 h, and treated with GES reagent (5 M guanidine thiocyanate, 100 mM EDTA, and 0.5% sarkosyl). The samples were further treated with ammonium acetate, purified with chloroform:isoamyl alcohol (24:1), and precipitated with ethanol. The precipitated DNA pellets were dissolved in 50 μl of TE buffer. The metagenomic DNA of *Wagashi* cheese samples was extracted as described earlier (Anihouvi et al., 2021) using the NucleoSpin^®^ Food (Macherey-Nagel GmbH&Co.) following the manufacturer's instructions. Qualitative (A260/280) and quantitative estimations of the extracted DNA of both food types were performed using a spectrophotometer (NanoDrop ND-1000, United States). The DNA was stored at −20°C for subsequent 16S rRNA gene amplicon sequencing.

### 2.3. Barcoded illumina MiSeq sequencing and data processing

Barcoded Illumina MiSeq amplicon sequencing was used for in-depth bacterial community structure analyses. Two independent approaches were performed for the spontaneously fermented milk products and traditional *Wagashi* cheese. For fermented milk products, the V4-V5 region of the 16S rRNA gene was targeted with the forward primer F563–577 (5′-AYTGGGYDTAAAGNG-3′) and reverse primer R924–907 (5′-CCGTCAATTCMTTTRAGT-3′) with barcodes for sample multiplexing as described earlier (Romi et al., 2015). The PCR-amplified DNA was purified using a QIAquick gel extraction kit (Qiagen, New Delhi, India) and quantified with a Qubit dsDNA BR Assay Kit in a Qubit 2.0 fluorometer (Invitrogen) for multiplexing in equimolar proportion. The DNA pool was sequenced on the Illumina MiSeq platform (Xcelris, Ahmedabad), and the sequence data were processed through the QIIME v1.8.0 bioinformatics pipeline (Caporaso et al., 2012) for adapter sequence removal, paired-end read generation, and sample de-multiplexing. A further MG-RAST pipeline was used at a 97% similarity threshold against the M5RNA database for the generation of OTU tables at different taxonomic levels. For the *Wagashi* cheese samples of Benin, the V1-V2 region of the 16S rRNA gene was targeted using the forward primer 28F (5′-GAGTTTGATCNTGGCTCAG−3′) and reverse primer 388R (5′-TGCTGCCTCCCGTAGGAGT-3′) with Illumina adapter and barcodes (Anihouvi et al., 2021). The target was PCR amplified in an ABI Verti-thermocycler, purified, pooled in equimolar proportion after quantification using a Qubit assay, and sequenced on Illumina MiSeq at RTL Genomics (Lubbock, TX, United States) as described earlier (Anihouvi et al., 2021). The sequence data were processed by PEAR sequence merger, USEARCH clustering and alignment algorithm, and UPARSE algorithm for OTU generation at different taxa levels.

### 2.4. Eubacterial-specific qPCR assay

A SYBR green-based qPCR assay targeting the SSU rRNA gene V3 region was performed for total bacterial load quantification. The PCR reaction containing 0.25 μM of each primer, with forward primer 338f 5′-ACTCCTACGGGAGGCAGCAG-3′ and reverse primer 518r 5′-ATTACCGCGGCTGCTGG-3′ (Ampe et al., 1999), and 1 × EXPRESS SYBR GreenER qPCR Supermix (Invitrogen), was used according to the manufacturer's instructions. The Applied Biosystems 7500 was used to carry out the PCR amplification at 95°C, 5 min, which consisted of 40 cycles of denaturation, annealing, and extension at 95°C for 15 s; 62°C for 30 s, and 68°C for 45 s, respectively (Keisam et al., 2016). A melt curve was generated for each assay from 60°C to 95°C using the default conditions to check the assay's specificity. The SSU rRNA gene (1 × 10^1^ to 1 × 10^8^ copies) derived from the strain type *Lactiplantibacillus plantarum* ATCC 8014 was used for the standard curve preparation.

### 2.5. Statistical analysis

The relative abundance data of the bacterial OTUs at the different taxonomic positions were used for statistical analysis. The significant differences in the relative abundance (%) of taxa between the study groups were calculated by Student's two-tailed *t*-test and ANOVA. Principal coordinate analysis (PCoA) was performed using the Bray-Curtis dissimilarity (PAST v3.22) (Hammer and Harper, 2008). The PERMANOVA test with 10,000 permutations was used to observe the significance of the difference in the bacterial taxa in the samples between two geographical regions and express it as a Bonferroni-corrected *p*-value. For calculating the alpha diversity indices (Chao species richness and Shannon diversity index) between two geographical regions, the compare_alpha_diversity.py script was used in the QIIME pipeline (Morris et al., 2014). The observed differences were visualized as boxplots using BoxPlotR (http://shiny.chemgrid.org/boxplotr/). The Wilcoxon test using “svDialogs” in the R package (v3.5.2) was conducted to show the significance of the difference in the bacterial species between two geographical regions and express it as a Benjamini-Hochberg (BH)-corrected *p*-value (Tuikhar et al., 2019). The hierarchically clustered heat map to show the species-level significant difference between two geographical regions was visualized using “gplots” in R. The OTU data, with a relative abundance of more than 0.1% and a significance of *p* > 0.001, was used for the heatmap generation.

### 2.6. Data availability

The sequence data associated with this study are available on the MG-RAST server: https://www.mg-rast.org/mgmain.html?mgpage=project&project=mgp87174 and MG-RAST ID mgp104874.

## 3. Results

### 3.1. Spontaneously fermented milk products from Northeast India and West Africa display distinct bacterial diversity

**Figure 1:**
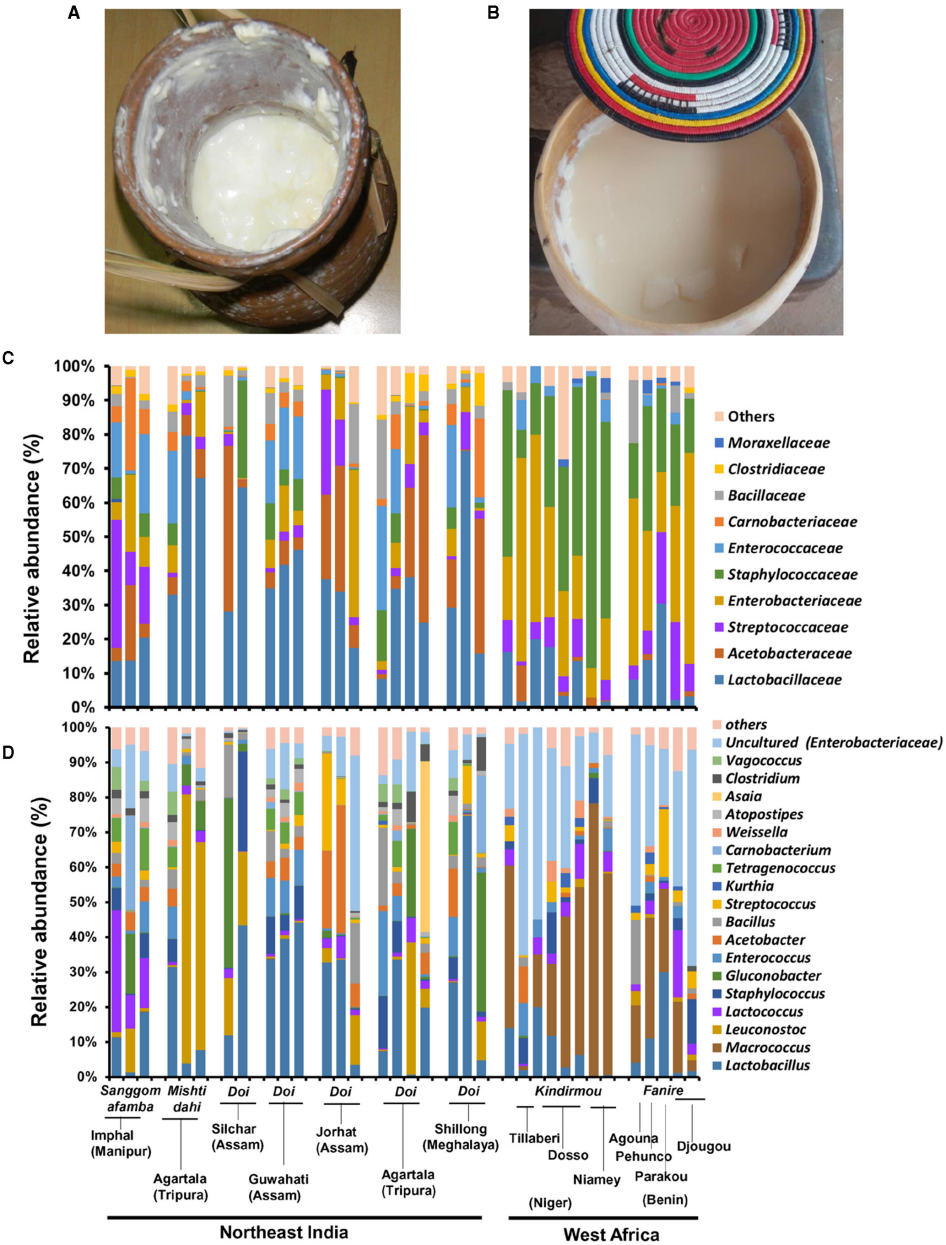
The bacterial community compositional difference in the spontaneously fermented milk products of Northeast India and West Africa. The spontaneously fermented milk products “*Doi”* in the traditional earthen pot **(A)** in Northeast India and “*Fanire”* in the traditional calabash vessel **(B)** in the Fulani camp of Benin are shown here. The taxon bar chart shows the family-level **(C)** and genus-level **(D)** differences in the relative abundance (%) of predominant bacteria present in the fermented milk products of Northeast India and West Africa. The sample details are available in Table 1.

We performed a cultivation-independent bacterial community analysis of spontaneously fermented milk products from Northeast India (*n* = 21, *Doi/Sangom afamba/Mishti dahi*) and West Africa (*n* = 13, *Kindrmou* of Niger and *Fanire* of Benin) by Illumina MiSeq amplicon sequencing of the 16S rRNA gene from the DNA of fermented milk samples. Bacillota (relative abundances of 72 and 61%) and Pseudomonadota (relative abundances of 17 and 3%) were the dominant bacterial phyla present in these naturally fermented milk products of India and West Africa, respectively. The mesophilic spontaneously fermented milk products of India analyzed in this study comprised *Lactobacillaceae* (34.7%), mainly *Lactobacillus* (*L. delbrueckii*) and *Leuconostoc* (*L. mesenteroides*); *Acetobacteraceae* (14.7%), mainly *Gluconobacter* and *Acetobacter* spp.; and *Streptococcaceae* (8.3%), mainly *Lactococcus* (*L. lactis*) and *Enterobacteriaceae* (8.3%, uncultured bacterium related to *Enterobacter* spp.) as core bacteria (Figure 1). However, the spontaneously fermented milk products of West Africa studied here contained *Staphylococcaceae* (34.6%), mainly *Macrococcus* (*Macrococcus caseolyticus*), *Enterobacteriaceae* (32.6%, uncultured *Enterobacter* sp.), *Streptococcaceae* (8.4%, *L. lactis*), and *Lactobacillaceae* (8%, *L. delbrueckii*) as the dominant bacteria.

**Figure 2:**
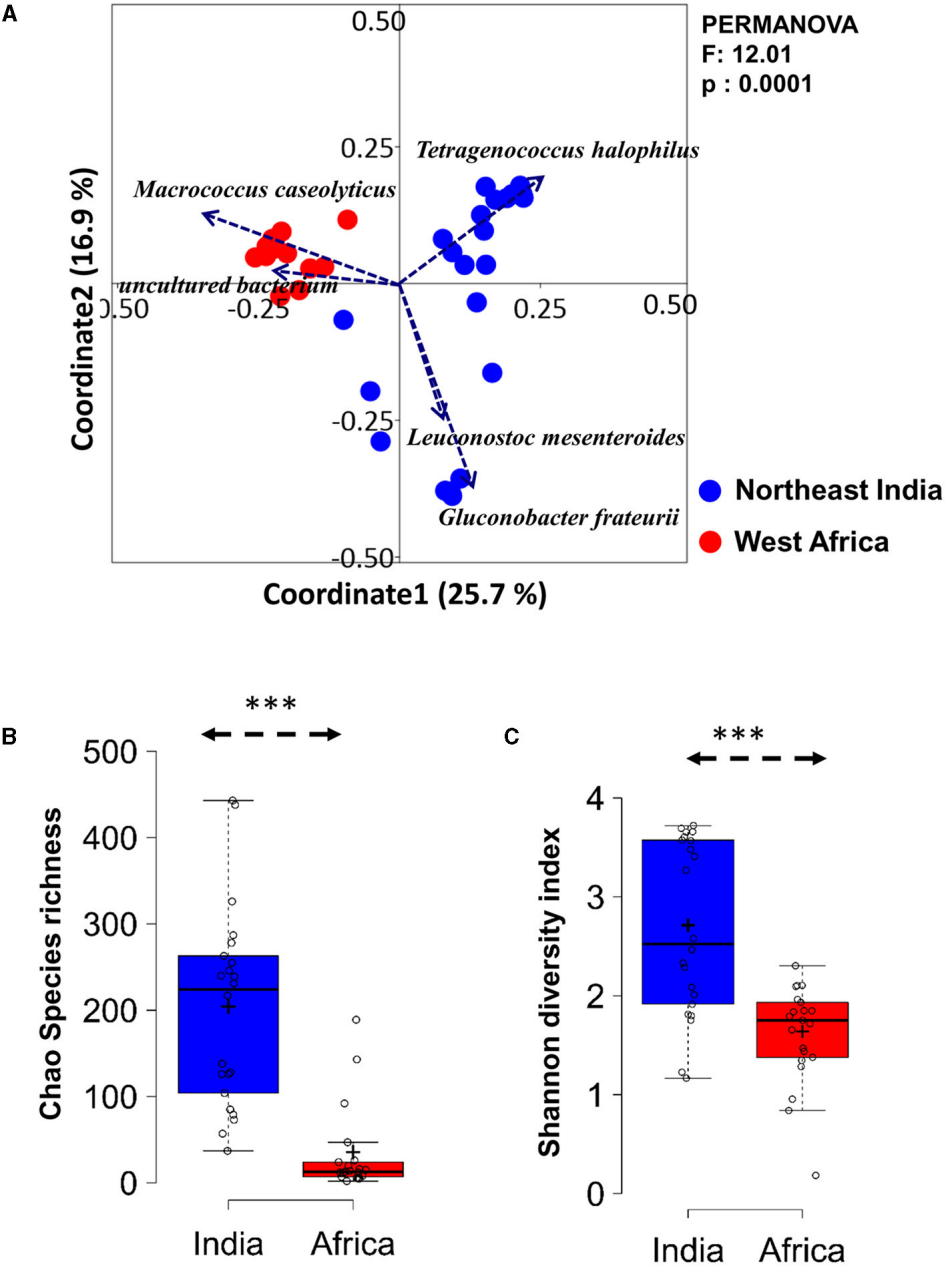
Differences in bacterial diversity in the spontaneously fermented milk products of Northeast India and West Africa. **(A)** PCoA biplot based on Bray-Curtis dissimilarity of the species-level OTUs shows a significant difference in the overall bacterial community structure between the spontaneously fermented milk products of the two continents. The significance of the difference is expressed as a Bonferroni-corrected *p*-value (*q* = 0.0001, *F* = 12.0, PERMANOVA). **(B, C)** The boxplot shows higher bacterial diversity in Indian fermented milk products (Chao species richness and Shannon diversity index) than in West African samples. The significance of the difference was calculated using Student's *t*-test and indicated as ***p<0.0001.

An unweighted principal coordinate analysis (PCoA) based on the Bray-Curtis distance matrix using species-level relative abundance showed a distinct separation of the fermented milk samples of India from West Africa (PERMANOVA, *p* < 0.0001) (Figure 2A). The PCoA biplot displayed that *M. caseolyticus*, uncultured *Enterobacteriaceae, L. mesenteroides, G. frateurii*, and *Tetragenococcus halophilus* were the key ecological drivers that shaped up the overall bacterial community structure difference in the fermented milk products of two geographical regions.

The fermented milk products of India had significantly higher bacterial species richness (Chao1) and diversity (Shannon index) than the West African products (*p* = 2.34E-07, Student's *t*-test, two-tailed) (Figure 2B). The total bacterial load of fermented milk samples analyzed using a eubacteria-specific qPCR assay resulted in a 9.47–11.02 log10 bacterial load per gram of the samples, without any significant difference between Indian and African samples.

**Figure 3:**
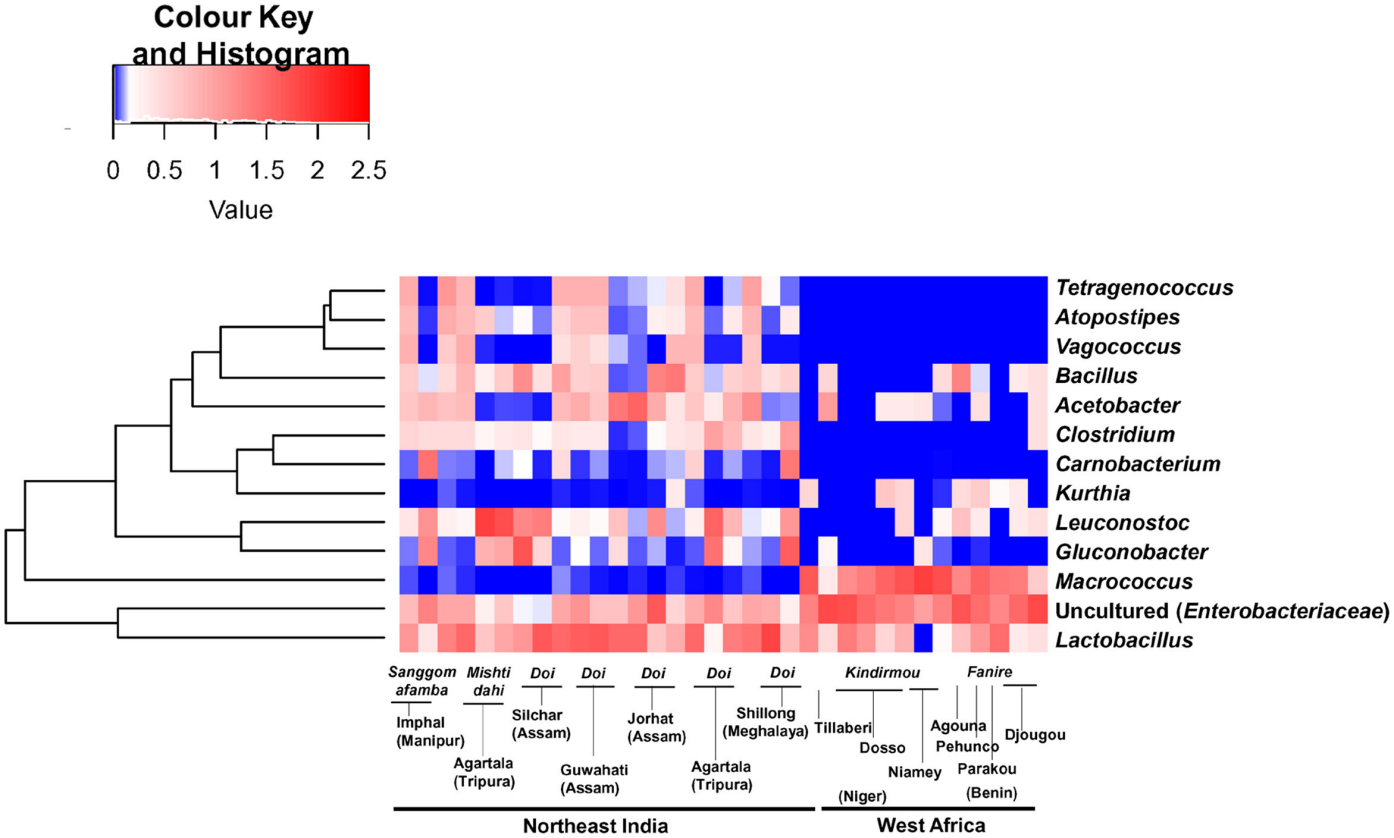
A hierarchically clustered heat map shows the significantly differing bacterial genera between the spontaneously fermented milk samples of Northeast India and West Africa. The bacterial genus with a relative abundance of more than 1% and significantly different between the two continents (Wilcoxon test, *q* < 0.001, BH corrected) is listed here. The abundance difference is shown as a red and blue colour gradient key.

While comparing the two geographical regions, *Acetobacteraceae* (*G. frateurii* and *Acetobacter* spp.) (*p* = 0.007) and *L. mesenteroides* (*p* = 0.014) were abundant in fermented milk samples from India, while *Staphylococcaceae* (*M. caseolyticus*) (*p* = 9.72 E-07) and uncultured *Enterobacteriaceae* were present abundantly in West African fermented milk samples (*p* = 1.23 E-05). In addition, *Tetragenococcus, Atopostipes*, and *Vagococcus* were present only in Indian fermented milk products (Figure 3). Among the beneficial Actinobacteria present in fermented milk products (Parker et al., 2018), *Bifidobacterium* was detected in only two samples from India. Within Indian samples, *Streptococcaceae* (21.4%, mainly *L. lactis*) were abundant (*p* = 0.014, Student's *t*-test, two-tailed) in the *Sangom afamba* samples, where backslopping was practiced with unpasteurised raw milk.

### 3.2. Bacterial community differences in the colored and uncoloured *Wagashi* cheese

**Figure 4:**
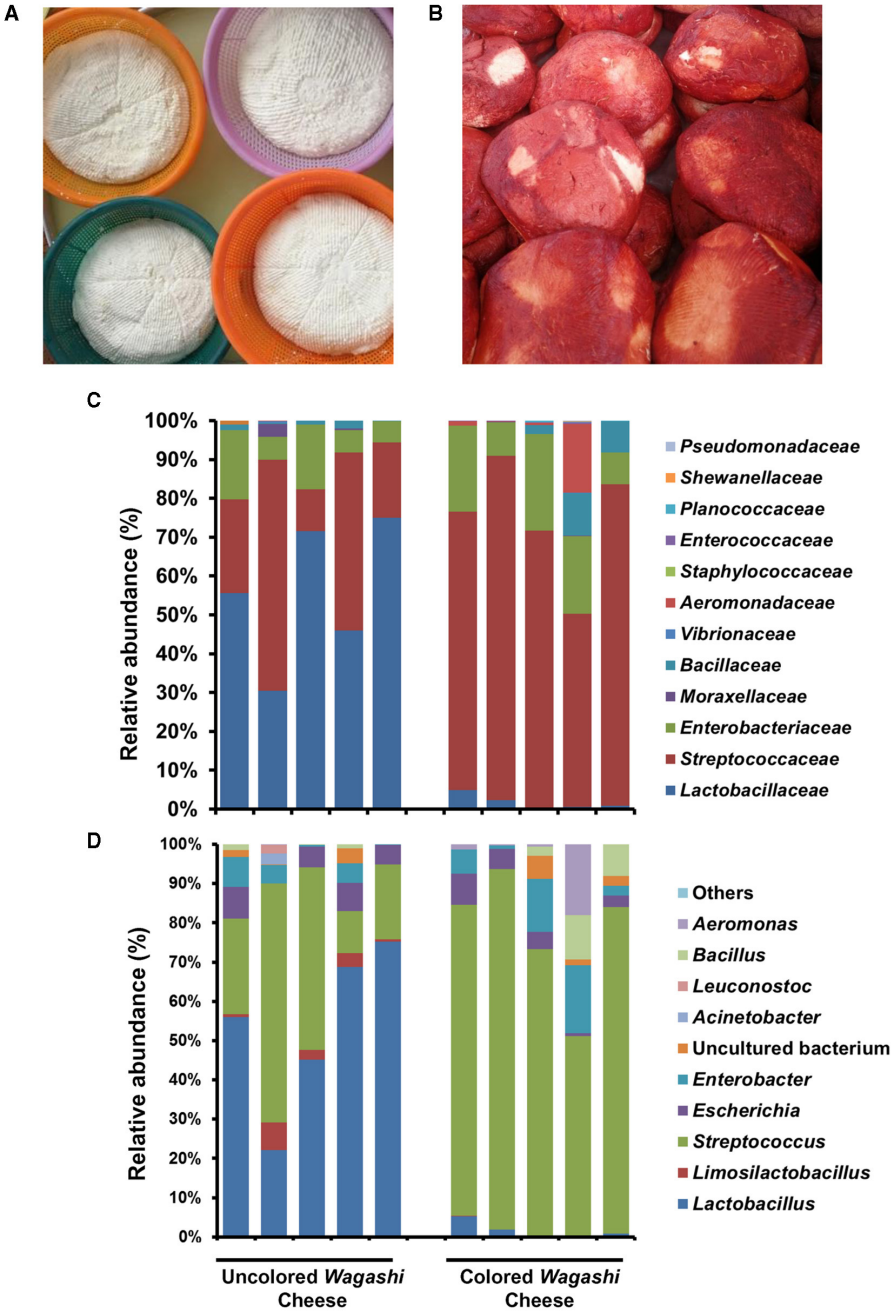
The difference in the overall bacterial community structure in the uncoloured **(A)** and coloured *Wagashi* cheese **(B)** marketed in Benin. The taxon bar chart shows the difference in the relative abundance (%) of predominant bacteria present in the uncoloured *Wagashi* cheese and coloured *Wagashi* cheese at the family level **(C)** and genera level **(D)**.

The bacterial community structure of uncoloured and colored *Wagashi* cheese of Benin (*n* = 10) prepared by spontaneous fermentation of boiled cow milk was analyzed by Illumina amplicon sequencing and compared with other reported traditional cheeses. At the phylum level, Bacillota (relative abundance of 85.1 and 76.2%) and Pseudomonadota (14.8 and 23.9%) were dominant in the uncoloured and colored *Wagashi* cheese samples, respectively. *Lactobacillaceae* (55.4%), mainly *Lactobacillus* (*L. delbrueckii*), and *Streptococcaceae* (31.9%), mainly *Streptococcus* spp., were dominant in the uncoloured *Wagashi* cheese. However, the colored *Wagashi* cheese was detected mainly with *Streptococcaceae* (72.9%) and low *Lactobacillaceae* (1.6%). Although no significant difference in the overall bacterial diversity (Chao species richness and Shannon diversity index) was visible between these two cheese types, we noticed a drastic change in the abundance of *Lactobacillus* (*L. delbrueckii, p* = 0.01, Students *t*-test, paired two-tailed) after soaking with Sorghum (*Sorghum bicolor*) leaf extract in the colored Wagashi cheese (Figure 4).

### 3.3. Safety of the spontaneously fermented milk products of Northeast India and Western Africa

In addition to the fermenting beneficial bacteria, the 16S rRNA gene amplicon sequencing-based in-depth analysis effectively detected the presence of several unwanted potential pathogens in the fermented milk samples. Our study detected *Clostridium* spp. in all the *Doi* samples up to 4% of relative abundance, particularly in the samples collected from Tripura and Meghalaya of Northeast India, whereas none of the West African fermented milk samples detected *Clostridium*. The *Wagashi* cheese samples from Benin contained *Streptococcus infantarius*, with up to 80% relative abundance in some samples. In addition, we found an uncultured *Enterobacteriaceae* bacterium with <96% similarity with the 16S rRNA sequence of *Enterobacter* spp. in West African and Indian fermented milk products. On average, *Kindrmou* and *Fanire* samples contained the uncultured bacterium related to *Enterobacter* with a 32% relative abundance, and *Wagashi* cheese samples had a 15% relative abundance of *Enterobacter*. In addition, *Wagashi* cheese contained, on average, more than 4% relative abundance of *Escherichia coli*. Among the members of *Staphylococcus*, we detected *Staphylococcus aureus* in West African fermented milk products and *Staphylococcus epidermis* in Indian *Doi* samples. While compared to the uncoloured Wagashi cheese, *Bacillus cereus* was abundantly present, with more than 5% average relative abundance in the colored, processed Wagashi cheese samples.

## 4. Discussion

Unlike thermophilic *yogurt* fermentation, in which *L. delbrueckii* and *S. thermophilus* dominate (Bokulich et al., 2015; Parker et al., 2018; de Melo Pereira et al., 2022), the mesophilic spontaneously fermented milk products of Northeast India comprise mainly *Lactococcus, Leuconostoc, Gluconobacter, Acetobacter*, and *Enterobacter*, in addition to *Lactobacillus*. Most of the NGS-based earlier studies reported the dominance of *Lactococcus* in naturally fermented milk products prepared by backslopping (Jans et al., 2017; Shangpliang et al., 2018; Gastrow et al., 2020; Moonga et al., 2020). Similarly, *L. mesenteroides*, which can tolerate high concentrations of sugar compared to other lactic acid bacteria (Fellows, 2017), was abundantly present in the *Mishti Dahi*, prepared by boiling milk with 10% sugar, and allowed mesophilic fermentation by backslopping (Mallappa et al., 2021).

The NGS-based earlier studies showed the dominance of *L. lactis, L. helveticus, L. mesenteroides*, and *Acetobacter* spp. in the *Dahi* prepared by backslopping in the high-altitude Himalayan region (Shangpliang et al., 2018; de Melo Pereira et al., 2022). At the same time, the *Dahi* prepared in southern India had *L. delbrueckii* as the dominant bacteria (Jayashree et al., 2013; Joishy et al., 2019; Mallappa et al., 2021). However, *Streptococcus* spp. and *Enterococcus* spp. were the most abundant in the *Dahi* samples of Bangladesh (Nahidul-Islam et al., 2018) and Bhutan (Shangpliang et al., 2017), respectively. *Enterococcus durans* predominates in the naturally fermented milk of cow and yak products of Arunachal Pradesh in India, such as mar, chhurpi, and churkam (Shangpliang and Tamang, 2021). Among the beneficial Actinobacteria reported in the fermented milk products (Parker et al., 2018), only two *Doi* samples were detected with *Bifidobacterium*.

On the contrary, the spontaneously fermented raw milk products of West Africa studied here contained *Macrococcus* and *Enterobacter* as the dominant bacteria. Unlike other species of *Staphylococcaceae, M. caseolyticus*, abundantly present in the fermented milk products of West Africa, is not considered a human pathogen and has been used as a starter culture for aroma and flavor production in several fermented foods (Ramos et al., 2021). The unique characteristic property of Fulani fermented milk products prepared in calabash containers may be linked with such bacterial associations (Groenenboom et al., 2020; Moonga et al., 2020; Fagbemigun et al., 2021). However, earlier NGS studies did not report *M. caseolyticus* as a primary bacterium in other African fermented milk products such as *Nono* (Fagbemigun et al., 2021) and *Mabisi* (Moonga et al., 2020). The bacterial diversity of fermented foods inferred from metagenomic studies differs depending on the method of DNA extraction used. In this study, we used an enzymatic lysis method that recovers a high bacterial diversity from fermented milk products (Keisam et al., 2016). We speculate the DNA extraction method adopted here may be one reason for the above differences, in addition to the environmental factors during the sampling (Zhao et al., 2021).

Earlier NGS analysis of similar spontaneously fermented unpasteurised cow milk “*Nono”* prepared in Nigeria by mesophilic fermentation in a calabash container without backslopping contained mainly *Lactobacillus* spp. and *Acetobacter* spp. (Fagbemigun et al., 2021). Another Zambian traditionally fermented milk, “*Mabisi*,” prepared similarly, was dominated by *L. lactis* (Groenenboom et al., 2020; Moonga et al., 2020). On the contrary, *S. thermophilus* and *L. helveticus*, reported worldwide as dominant bacteria in several naturally fermented milk products (Oki et al., 2014; Bokulich et al., 2015; Shangpliang et al., 2018; Peng et al., 2021; Yu et al., 2021), were rare (<2% relative abundance) in the spontaneously fermented milk products of this study. The mesophilic spontaneous fermentation, without backslopping, might be the reason for such a difference in the predominant bacterial communities. Another observation of total bacterial load analyzed by culture-independent qPCR assay is much higher than those reports based on the cultivation-dependent analysis of similar fermented milk products with 6.0–9.0 log 10 population load per gram (Dewan and Tamang, 2007; Shangpliang et al., 2017), which support the possible presence of unculturable strains of dominant bacterial species in natural milk fermentation.

The presence of *Lactobacillus* (*L. delbrueckii*) and *Streptococcus* as dominant bacteria in the uncoloured *Wagashi* cheese is similar to the Mexican Poro Cheese (Aldrete-Tapia et al., 2014) and traditional cheeses of China, Mongolia, and Russia (Zhong et al., 2016; Zhao et al., 2021). The drastic change in the main bacterial abundance in the colored Wagashi cheese is linked with the Sorghum leaf extract used for coloring and further drying. Sorghum leaf extract mainly contains apigeninidin pigment and phenolic compounds, normally used for prolonging shelf life (Akogou et al., 2018). The antimicrobial activity of sorghum leaf extract (Akogou et al., 2018; Schnur et al., 2021) and further processing, such as drying, may have favored *Streptococcus* spp. Contrary to the cheese types produced worldwide from cow milk through natural fermentation, *L. lactis* and *L. mesenteroides* (Delcenserie et al., 2014; Shangpliang et al., 2017; Rocha et al., 2021; Sun and D'Amico, 2021; Zhang et al., 2021) were not predominant in *Wagashi* cheese, while *Streptococcus* spp. dominated. The traditional production method of curdling with proteolytic leaf extract of *Calotrophis procera* and thermophilic incubation at 60–70°C (Akogou et al., 2018; Sessou et al., 2019) might have favored *Streptococcus* spp. in *Wagashi* cheese of Benin.

*Clostridium* in naturally fermented milk products is a safety concern in Northeast India, where the Indian Council of Medical Research (ICMR) has already reported high incidences of foodborne diseases and intestinal infections (ICMR Report, 2017). Earlier NGS-based studies also detected a similar presence of *Clostridium* in the *Dahi* samples of Bangladesh (Nahidul-Islam et al., 2018). Our study detected *Clostridium* in the *Doi* samples collected from Tripura and Meghalaya, which are Indian states bordering Bangladesh. Several earlier reports also showed the presence of *Clostridium* in other fermented foods (particularly in fermented pork, fish, and soybean products) marketed in Northeast India (De Mandal et al., 2018; Keisam et al., 2019; Deka et al., 2021).

The *Wagashi* cheese samples from Benin contained *Streptococcus infantarius*, possibly related to *S. infantarius* subsp. *infantarius* (Sii), belonging to the *Streptococcus bovis*/*Streptococcus equinus* complex (SBEEC), which has recently been reported as an emerging infectious foodborne pathogen in African countries (Jans et al., 2017). Attempts on selective isolation and characterization of an uncultured *Enterobacteriaceae* bacterium found in West African and Indian fermented milk products could result in novel species. The earlier NGS studies also reported the dominance of *Enterobacter* spp. in the African naturally fermented milk *Mabisi* (Moonga et al., 2020) and *Kefir* of Turkey (Dertli and Çon, 2017). Similar to our results, several reports based on 16S rRNA gene sequencing account for the presence of *E. coli* in traditional cheese produced by natural fermentation (Fuka et al., 2013).

Most West African fermented milk products are prepared from raw, unpasteurised milk by spontaneous fermentation (Leone et al., 2022). Usually, unpasteurised raw milk contains *Staphylococcaceae* members abundantly (Joishy et al., 2019; Sun and D'Amico, 2021), which supports the dominance of *M. caseolyticus* in spontaneously fermented raw cow milk in West African countries. Moreover, several traditionally fermented milk products have been reported to contain *M. caseolyticus* (Fuka et al., 2013). Few other studies have detected *M. caseolyticus* in foods of animal origin (Aldrete-Tapia et al., 2014; Ramos et al., 2021). However, it is not prioritized as a human pathogen and has been used as a starter culture in several fermented foods (Ramos et al., 2021). The traditional milk curdling with proteolytic leaf extract of *Calotrophis procera*, thermophilic incubation at 60–70°C, and further processing by colouration and drying might have favored the *B. cereus* growth in the colored Wagashi cheese. Lowering the moisture content by drying and adding 3% NaCl to the colored Wagashi cheese would reduce the presence of *B. cereus* (Raevuori and Genigeorgis, 1975; Rukure and Bester, 2001). These findings will allow us to understand the safety issues related to spontaneous milk fermentation and to develop strategies to overcome them by identifying the critical control points of pathogen entry. Moreover, technological intervention by designing the starter culture consortiums for a controlled fermentation of boiled or pasteurized milk will improve the safety and quality of the fermented milk products in both regions studied (Agyei et al., 2020; Mallappa et al., 2021; de Melo Pereira et al., 2022).

Natural milk fermentation is highly reproducible across geographical regions under similar processes and conditions (Wolfe et al., 2014). Such substrate- and condition-specific adaptive evolution of microbiota is responsible for the unique properties of different fermented foods (Tamang et al., 2021). In our study, natural mesophilic fermentation of unpasteurised cow milk without backslopping in two geographically separated regions shaped different bacterial community structures. In contrast to the thermophilic spontaneous milk fermentation by backslopping commonly practiced worldwide, the mesophilic milk fermentation without backslopping favored a combination of lactic acid bacteria (*Lactobacillus* and *Lactococcus)* and acetic acid bacteria (*Gluconobacter* and *Acetobacter*) in Indian *Doi* samples. Meanwhile, *Macrococcus* and an uncultured bacterium of *Enterobacteriaceae* were found in Fanire and Kindirmou samples of Benin and Niger, respectively. Such differences in the microbiota evolving during natural fermentation may relate to the microbiota of raw cow milk used and the indigenous production process (in traditional earthen pots or calabash containers) in practice in both regions. Moreover, we report for the first time the bacterial communities of Wagashi cheese in Benin and their differences during processing (coloring). This understanding will allow us to implement controlled production through technological intervention by designing the starter culture consortiums, which improve the safety and quality of the traditional fermented milk and cheese products in India and Africa, where natural fermentation is in practice.

## Data availability statement

The data presented in the study are deposited in the MG-RAST repository, MG-RAST accession numbers are mgp104874 and mgp87174. The sequence data associated with this study are available on the MG-RAST server: https://www.mg-rast.org/mgmain.html?mgpage=project&project=mgp87174 and MG-RAST ID mgp104874.

## Author contributions

KJ, PS, and SK were involved in conceiving the research idea and designing the work plan. The experiments were performed by SK (Indian fermented milk), PS, MG (fermented milk of Benin and Niger), and GK (*Wagashi* cheese of Benin). KJ and SK analyzed the data and interpreted the results. KJ and PS wrote the manuscript. GK, SF, and JM revised the manuscript. All authors contributed to the article and approved the submitted version.
